# Medical Items

**Published:** 1908-09

**Authors:** 


### MEDICAL ITEMS

---

PSEUDOANEMIA: Do not forget that not every anemic-looking patient has anemia, a lack of red blood corpuscles. The diathetic state known as lithemia very often induces such a contraction of the peripheral circulation as to produce a condition of palor that may be mistaken for anemia. The condition, however, is one of ischemia instead of anemia, and does not call for iron. The therapeutic indications are to overcome the underlying lithemia, and for this purpose there is no remedy superior to Alkalithia, made by the Keasbey & Mattison Co., Ambler, Pa.

---

Measured by every standard of purity, Peacock's Bromides is never successfully imitated. This is why it is necessary for the physician to see that the genuine is dispensed. He thus insures his results in all bromide treatment, particularly in this instances in which the prolonged use of the salts seems indicated and desirable. Neurologists have called especial attention to this featurs of the preparation Peacock's Bromides, and therefore it is the severest therapeutic test to which the Bromides can be put, and there is no doubt that purity is of great importance in such cases.

---

CARDIAC TONIC.—"I have prescribed Cactina Pillets in a number of cases of heart trouble and find them a reliable cardiac tonic, especially in weak heart with small, frequent intermittent pulse. They are a specific in functional heart trouble."—R. A. Clopton, M. D., Milan, Tenn.

